# An economic model of advance care planning in Australia: a cost-effective way to respect patient choice

**DOI:** 10.1186/s12913-017-2748-4

**Published:** 2017-12-01

**Authors:** Kim-Huong Nguyen, Marcus Sellars, Meera Agar, Sue Kurrle, Adele Kelly, Tracy Comans

**Affiliations:** 1 Center for Applied Health Economics, Griffith University, Brisbane, Queensland Australia; 2 NHMRC Cognitive Decline Partnership Centre, the University of Sydney, Sydney, New South Wales Australia; 3 Advance Care Planning Australia, Austin Health, Melbourne, Victoria Australia; 4 Kolling Institute, Northern Clinical School, Faculty of Medicine, The University of Sydney, Sydney, New South Wales Australia; 5 University of Technology Sydney, Sydney, New South Wales Australia; 6 University of New South Wales, Sydney, Australia; 7 Ingham Institute of Applied Medical Research, Sydney, New South Wales Australia; 8 South West Sydney Local Health District, Sydney, New South Wales Australia; 9 Northern Clinical School, University of Sydney, Sydney, New South Wales Australia; 10 HammondCare, Sydney, New South Wales Australia; 11 Center for Health Service Research, Faculty of Medicine, The University of Queensland, Brisbane, Queensland Australia; 12 Metro North Hospital and Health Service District, Brisbane, Queensland Australia

**Keywords:** Advance care planning, Cost effectiveness, Economic evaluation, Markov model, End of life, Dementia

## Abstract

**Background:**

Advance care planning (ACP) is a process of planning for future health and personal care. A person’s values and preferences are made known so that they can guide decision making at a future time when that person cannot make or communicate his or her decisions. This is particularly relevant for people with dementia because their ability to make decisions progressively deteriorates over time. This study aims to evaluate the cost-effectiveness of delivering a nationwide ACP program within the Australian primary care setting.

**Methods:**

A decision analytic model was developed to identify the costs and outcomes of an ACP program for people aged 65+ years who were at risk of developing dementia. Inputs for the model was sourced and estimated from the literature. The reliability of the results was thoroughly tested in sensitivity analyses.

**Results:**

The results showed that, compared to usual care, a nationwide ACP program for people aged 65+ years who were at risk of dementia would be cost-effective. However, the results only hold if ACP completion is higher than 50% and adherence to ACP wishes is above 75%.

**Conclusions:**

A nationwide ACP program in the primary care setting is a cost-effective or cost-saving intervention compared to usual care in a population at-risk of developing dementia. Cost savings are generated from providing treatment and care that is consistent with patient preferences, resulting in fewer hospitalisations and less-intensive care at end-of-life.

**Electronic supplementary material:**

The online version of this article (10.1186/s12913-017-2748-4) contains supplementary material, which is available to authorized users.

## What is already known about the topic?

- Advance care planning (ACP) empowers patients, with the support of their caregivers, to consider and communicate their current and future treatment goals in the context of their own preferences and values. However, the uptake ACP in older people and people with dementia remains low.
- Known benefits of ACP include reduced stress and anxiety for the surviving family members and an increased likelihood that patient wishes will be known and respected at end-of-life. Preliminary research also suggests that ACP may reduce costs or be cost-neutral at end-of-life.
- Studies estimating the economic impact of ACP are scarce and no studies have systematically identified costs and outcomes, or the potential trade-off between these factors.

## What this paper adds?

- This is the first study to use a Markov cohort model to estimate the cost-effectiveness of a nationwide ACP program in a primary care setting for people aged 65+ years who are at risk of dementia.
- This study found that providing an nationwide ACP intervention in a primary care setting for people aged 65+ before they develop dementia, in a series of consultations lasting between 30 and 90 min each, is a cost-effective strategy compared to the current situation (without the nationwide intervention).
- The study identified three interdependent determinants of a successful ACP program: uptake rate, compliance, and end-of-life choices. The cost-effectiveness cannot be substantiated if high ACP uptake is accompanied by a low compliance with ACP, or if end-of-life hospitalisation continues to be the dominant choice due to a lack of well-supported alternatives in the community.

## Implications for practice, theory or policy

- The study has important implications for the design and implementation of a national ACP policy, both in Australia and internationally. It highlighted the importance of a systematic approach along the continuum of care and a flexible ACP design to accommodate different life situations.

## Background

Empirical studies show that healthcare costs increase exponentially at end of life [1–4], but increased spending may not improve quality of care [5]. In addition, aggressive medical treatment at the end of life may be inconsistent with the care preferences of the patient [6] or prove to be unnecessary [7]. In Australia, approximately 80% of adults will be admitted to hospital in the last year of their life, with more than 30% spending more than 30 days in hospital, 55% dying in hospital, 12% attending the intensive care unit and only 24% receiving palliative care [8].

Advance care planning (ACP) is a process that explores a person’s values, beliefs and preferences regarding future health and personal care to guide medical decision making at a future time when that person can no longer communicate his or her decisions. This process usually involves the patient, family members, other important persons and healthcare providers [9]. ACP has been shown to improve the likelihood that doctors and family members will know and comply with the patient’s wishes [9–11], increase hospice and palliative care use, decrease inappropriate life-sustaining treatments and hospital admissions, improve patient and family satisfaction with care, as well as reduce stress, anxiety and depression in surviving relatives [10, 12–14]. ACP is particularly important in the dementia context because the cognition and decision-making capacity of individuals with dementia becomes increasingly limited as the disease progresses, and ACP becomes the sole vehicle to foster autonomous decision making [15].

Because ACP increases the likelihood that the medical treatment wishes of the patient will be known and respected, it also has an impact on the cost of care at end–of-life. Two recent systematic reviews examining the effects of ACP on cost of end-of-life care were identified. Klingler et al. (2015) showed that facilitated ACP had the potential to reduce net costs of care [16], while Dixon et al. (2015) found associations between ACP and healthcare savings for people living with dementia in the community, and for people living in areas with high end-of-life care spending [17]. However, the authors did not systematically identify both costs and outcomes, or the potential trade-off between these factors.

This study aims to overcome the limitations of past economic evaluations of ACP. We developed an economic model to assess the cost-effectiveness of an ACP intervention in a cohort of older people (aged 65+ years) who were at risk of cognitive decline in the context of the Australian healthcare system. The Markov modelling technique was used to simulate a decision problem involving risk over time (e.g. progression of dementia), when the timing of events is important (e.g. introducing ACP before loss of ability to communicate in people with moderate to severe dementia) and when important events may repeat (e.g. treatment or care for episodes of illness). It can mimic the clinical pathway and allows the capture of costs and outcomes associated with important events over time. Markov models have been widely used in economic evaluations of healthcare interventions to inform funding decisions.

We hypothesised that a nationwide ACP program delivered to an Australian population aged 65+ years who are at risk of developing dementia would:

(i) increase the rate of ACP completion
(ii) increase the likelihood that end-of-life wishes will be known and respected, and
(iii) result in more efficient use of healthcare resources by reducing the use of unnecessary healthcare interventions near the end of life.

## Methods

### Overview

A life-time health state transition model was used to combine data on the healthcare costs and health outcomes of two scenarios: (i) with a nationwide ACP program in the primary care setting, and (ii) current situation. The study population were adults aged 65+ years. It is known that while dementia is not a normal part of aging, age itself is the highest risk factor of developing dementia. Using the Australian health system perspective, the key outcome was the incremental cost per quality-adjusted life year (QALY), which represents the additional cost of an ACP program per additional QALY gained (if any) compared with the current situation. Both costs and outcomes were discounted at 5% in the base case and at 2% and 7% in the sensitivity analyses. The reliability of the results was tested in sensitivity analyses, which included threshold analyses and second order probabilistic sensitivity analyses with 5000 replications.

### Scenarios

#### Current situation

The Australian healthcare system does not currently have a consistent systematic approach to ACP. ACP programs are provided by some community services, residential aged care facilities and tertiary hospitals. Some primary care providers also support individuals to complete advance care directives (ACDs). Models vary from employing dedicated ACP facilitators to training existing clinicians to conduct ACP in addition to their role; thus, the service quality provided to patients can also vary. The current uptake of ACP in people aged 65+ years in Australia does not exceed 15% [9, 18, 19].

#### Introduction of a nationwide ACP intervention in the primary care setting

We propose a nationwide primary care program to provide ACP to people aged 65+ years. The program has a relatively flexible design so the ACP discussions can be conducted in various settings (e.g. general practitioners’ offices, nurse-led medical centres, residential care or homes). Once individuals reach the target age (65 years), their doctors, nurses or dedicated ACP facilitators will initiate the ACP discussion by providing relevant materials and following up for further development, or referring them to the most suitable settings. It is estimated that the whole ACP process, from initial discussion to the completion of an ACD, will be undertaken over two to four face-to-face consultations. An additional review of the ACD should occur at critical times, such as when individuals are diagnosed with dementia or a terminal illness, or other life-changing circumstances. The ACD should be shared between the individuals’ families and the health system (i.e. general practitioners, specialists and hospitals).

Elements of this proposed intervention were based on extensive research which indicated that primary care settings are an ideal platform for an ACP program [20, 21], and that ACP development should involve repeated consultations and discussions with the individuals, their families and their healthcare providers [11].

### Model structure

**Fig. 1:**
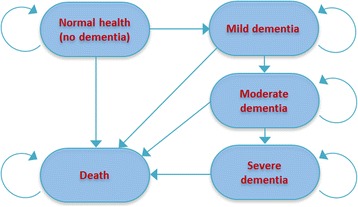
Health state transition diagram

A life-time Markov cohort model with a one-year cycle was constructed and analysed in TreeAge Pro 2015 software. Five health states were used to account for the development and progression of dementia and for end-of-life (see Fig. 1). Dementia stages include mild, moderate and severe, based on the Clinical Dementia Rating classification and previous modelling approaches [22–24].

Older people (aged 65+ years) entered the model and moved between the various health states according to dementia progression and general health deterioration. Both the probabilities of developing dementia and the mortality risk were age dependent, while the probabilities of disease progression (from mild to moderate, from moderate to severe) and the preference for end-of-life hospitalisation were constant over time. Since dementia is a non-reversible condition, individuals can only move from mild to moderate to severe, but not the reverse. Because disease states other than dementia can also result in loss of capacity, the model dictated that at any point in time, an individual could fall very ill and move to end-of-life care before they died.

In the model, an individual could engage in the ACP process while he/she did not have dementia. This assumption ensured that ACP was commenced well before the dementia progression limited an individual’s ability to fully engage in the process of eliciting their preferences and wishes [25–27]. The ACD, once completed, would be reviewed when the individual was first diagnosed with dementia or another terminal illness. A final review of the document would occur when the individual enters the end-of-life period, where the ACD might be discussed, revised or followed up by the family and medical professionals.

### Data input and resources

Since we focused on the impact of ACP on medical decisions at the end-of-life, both scenarios incorporated an identical likelihood of dying, risk of illness and developing dementia, and associated costs. The main differences related to the coverage of ACP; cost of the ACP program; the end-of-life care cost; and quality of life, including the disutility associated with the ACP non-compliance, or unknown preferences of the patients due to the absence of an ACD or surrogate decision maker. While most inputs were constant, the risk of developing dementia, all-cause mortality risk and hospitalisation costs increased with age. For the base case, we conservatively assumed that the quality of life and wellbeing of individuals would not be reduced when their wishes (specified in their ACD) were not respected (compared to the scenario where their wishes were fully respected). In reality, not having preferences and wishes honoured is likely to bring distress and lower wellbeing for individuals (represented as disutility in health economics).

To identify relevant evidence to populate the model, the Medline database and grey literature were searched. The search strategy used key words (and their combinations) such as advance care plan, advance care directive, dementia, and Alzheimer’s disease. A manual search of the references of each identified article of interest was also completed for further information. Other sources of information included national epidemiological studies and costing reports. All costs were calculated in 2015 Australian dollars. Detailed descriptions and assumptions of the data and their sources are presented in the Additional file 1: and the summary in Tables S1 and S2.

## Results

**Fig. 2:**
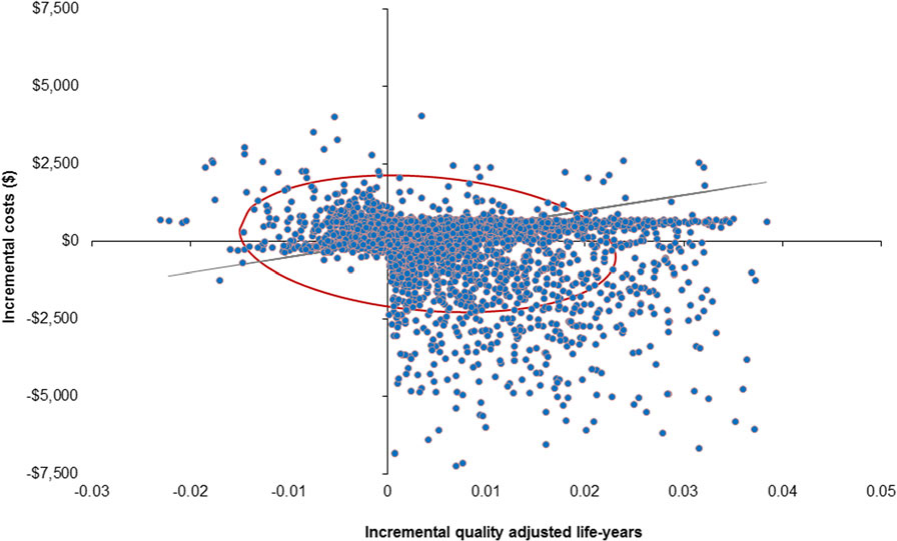
Probabilistic sensitivity analysis (Monte Carlo simulation with 5000 draws and willingness-to-pay of AUD 50,000)

The base case results and selected sensitivity analyses are presented in Table 1 and Fig. 2.

**Table 1:** Base case results and selected sensitivity analyses

Scenarios	Current situation	ACP program	Incremental cost ($)	Conclusion
Base case	6749	6682	−67	Cost effective
Starting age = 75 years (base case = 65 years)	7250	7160	−90	Cost effective
End-of-life non-hospitalisation cost = 70% hospitalisation cost (base case = 60%)	7194	7216	22	Not cost effective
ACP coverage, with ACP program = 30% (base case = 50%)	6749	6765	−16	Cost effective
ACP compliance rate, with ACP program = 60% (base case = 86%)	6816	6913	97	Not cost effective
Choose to die in hospital, with ACP program = 60% (base case = 15%)	7324	7563	239	Not cost effective
ACP program of 6 visits, excluding revision visits (base case = 4 visits, excluding revision visits)	6749	6754	5	Not cost effective

*Abbreviation: ACP* advance care planning

Note: We do not present the incremental cost effectiveness ratio (ICER) here because we conservatively assumed zero disutility associated with not having an end-of-life preference followed, which leads to same QALYs accrued for both scenarios. Instead, incremental costs are presented

### Base case findings

The base case assumed (i) the starting age for having an ACP was 65 years, and (ii) a 50% uptake rate of ACP should the program be introduced. The results showed that over a lifetime, compared to the current situation, a nationwide ACP program was less costly while achieving the same QALY outcome. The cost-saving came from the lower rate of hospital-based end-of-life care for patients who had an ACP. Patients in both scenarios (ACP program versus current situation) accrued the same QALYs because we conservatively assumed no quality of life difference associated with not having an end-of-life preference followed (i.e. zero disutility weight) [9, 28, 29]. If any disutility was assumed, ACP became a dominant option: cheaper and more effective.

### Sensitivity analyses

Extensive sensitivity analyses, including threshold analyses, were conducted on the key parameters to assess the likelihood of ACP remaining cost effective. The result was highly sensitive to several key parameters (see Table 1): ACP completion and compliance rates; and dying choice (hospital versus non-hospital settings). The probability sensitivity analysis of 5000 Monte Carlo replications highlighted that there was a 50–50 chance that a nationwide ACP program would be cost effective (see Fig. 2) due to high uncertainty around the key parameters.

#### Advance care planning completion rate

A breakeven point was identified for the completion rate in order for ACP to remain cost saving or neutral. Our model indicated that even at a low coverage (26%), ACP was still a cost-saving strategy if the majority of individuals prefer end-of-life care outside hospitals and if their wishes are respected.

#### Compliance rate

Compliance is the likelihood of the ACP being respected and followed. Our model predicted that if the compliance rate dropped below 75% then the ACP program was no longer cost effective. Additionally, with a moderate coverage (below 40%) and high hospitalisation rate for end-of-life care, even a high compliance rate was not sufficient to make the ACP program a good investment.

#### End-of-life cost

There is a consensus that hospital-based end-of-life care is more expensive than the alternatives such as home care, residential aged care facilities and hospice facilities [30]. However, little comparative data is available that quantifies the cost differences, especially for end-of-life care at home. International studies that are available might not be directly applicable to the Australian healthcare system due to health system differences [30–32]. In our base case, we started with an estimate that patients who did not die in hospital had roughly 40% less costs than those who died in hospital. The lower the former cost, the more cost-saving the ACP program would be. However, if the actual cost for dying outside of hospital rose to approximately 70% of cost of dying in hospital, the savings from unnecessary hospitalisation were insufficient to outweigh the cost of providing ACP.

#### Place of death

Past research showed that the majority of people would prefer to receive end-of-life care and die outside of hospital [33], and individuals who participated in ACP were significantly less likely to die in hospital [33–36]. We tested this proportion in a threshold analysis and found that if 60% of patients with an ACP wanted to receive end-of-life care in hospital, then the ACP program was no longer cost saving. This proportion was unlikely as surveys estimate that 60–70% of Australians would prefer to die at home [33, 37].

#### Cost of ACP

A more comprehensive ACP program might have higher costs than what is anticipated in our model. It has been suggested that an effective and systematic approach to ACP should include personalised discussion about life values and preferences for end-of-life care [38]. These discussions will occur over several visits, facilitated by paid and well-trained facilitators, and with active participation of informed surrogates. These visits can also be supported by oral, written and video recorded information, which will require more systematic and repeated training of the facilitators [38]. Another component of a comprehensive ACP program might include legal requirements for the dissemination of the ACP document amongst families and medical institutions. Our model indicated that if the cost per individual ACP reaches $850 (equivalent to seven visits) then the program is no longer cost effective. Nonetheless, this scenario is unlikely because individual ACP has been provided successfully in Australia through group information sessions followed by 1–2 visits by trained ACP facilitators, which costs less than AU$250. At this cost, the program is more likely to be cost-effective than the base case scenario.

## Discussion

This is the first dementia-specific study to evaluate the cost-effectiveness of ACP in a simulated population using the Markov modelling technique. These findings demonstrate that providing a nationwide ACP intervention comprised of three to four consultations (30–90 min each) for people aged 65+ years, who are at risk of dementia, is cost effective, provided that the ACP uptake and compliance with end-of-life wishes rates are at least 50% and 75%, respectively. Whilst we acknowledge that cost savings should not be the main aim of introducing a wider ACP policy, our model showed that a secondary positive outcome was the reduction in healthcare expenditure, making those resources available to other patients and population health interventions.

In line with the concept that ACP should be initiated ‘earlier rather than later’ in people with dementia, before the loss of capacity and ability to communicate occurs, our model indicated cost-effectiveness diminished when ACP was introduced later in life. This finding is not surprising as ACP discussion may be less feasible with an individual with moderate or severe dementia. For example, a systematic review examining the effectiveness of conducting ACP in people with dementia found that the majority of participants did not have the capacity to complete an ACP discussion in the nursing home setting and thus had to defer the decision-making responsibility to surrogates [34].

Our model showed that the economic benefit of a nationwide ACP program was dependent on a moderate uptake rate and a high compliance rate in an environment where dying outside hospital was a preferred choice. We found that an uptake rate as low as 26% was still cost saving if the majority of individuals preferred end-of-life care outside hospitals and if their wishes were respected. It is noted that the ACD is legally binding under statute legislation throughout most of Australia and under the Common Law in New South Wales and Tasmania, thus a high compliance rate (given a moderate-to-high uptake rate) is warranted. Additionally, if a national ACP program were to be introduced, it should contain an education component for both health care providers and community members to ensure that they understand that ACP compliance is not optional. Therefore, a critical consideration here is whether or not the demand for high quality palliative care services, both at home and in the hospice setting, can be met and, therefore, whether or not people’s wishes are feasible at the end of life.

Dying in hospital facilities and the use of life-sustaining measures are individual preferences that should be fully respected. However, delayed end-of-life decision-making usually leads to unwanted and/or unwarranted aggressive life-sustaining measures being instigated for those approaching end of life, including those who are imminently dying. Unwanted heroic measures have been shown to negatively influence quality of life in the week before death [37, 39], which may explain why most people prefer to die at home rather than in admitted inpatient settings [33]. This may be especially relevant for people in the advanced stages of dementia due to the prospect of imminent death, where caring and comforting, rather than curing, are needed. People with a preference to die at their residence (e.g. home or aged care facility) will require their carers and homes to be sufficiently supported and equipped to provide end-of-life care. Assessment of the current and potential risks for emergency and inpatient admission, and formulating a plan of action, may help prevent unwanted hospitalisation when a crisis or other intense situation occurs.

The ability to fully realise the benefits demonstrated in the modelling is dependent on accessible high quality community and palliative care services. Without access to support at home, it is likely that people at end of life will end up dying in hospital despite their expressed wishes. Our model suggested that an additional 15% of older people would die outside of hospital if this model of ACP was fully implemented. It is unlikely that current services would have capacity to care for these additional people at home and implementing this model of ACP in Australia would necessarily require an additional investment in community services by government and/or a realignment of funding from hospital care to community and residential aged care facilities. It is noted by the Productivity Commission [37] that approximately 70% Australians preferred to die at home but less than 10% were able to do so, and 20% of older people living in residential aged care facilities died in hospital due to a lack of palliative care expertise and qualified staff.

A complex multi-faceted ACP intervention is more likely to increase compliance with individuals’ end-of-life wishes [11]. The ACP program proposed in our paper is based on a series of consultations in the primary care setting, which is often considered an effective setting for ACP. First, general practitioners and other community-based health professionals are in an ideal position to facilitate ACP because they have an ongoing relationship with the patient and often understand his/her healthcare needs [20]. It is also recognised that people are more empowered in primary care than in other healthcare settings [21]. Second, community dwellers are still generally in good health and more likely to have decision-making capacity. As noted in Robinson et al. (2012) [34], residential aged care settings might be too late for starting the ACP journey as many residents have lost their decision-making capacity.

Furthermore, recent best-practice recommendations (Draft Recommendation 4.3 from the Productivity Commission’s Draft Report of June 2017 [37]) suggest the Australian Government should promote and aim to increase the uptake of ACP in primary care by a) requiring the general practitioner to introduce the concept of ACP and b) introducing a new Medicare item number to fund practice nurses to facilitate ACP. If such initiatives were implemented nationally we believe the feasibility of meeting the ACP uptake and compliance rates required in our model would be met and, thus, increase the likelihood of ACP being a cost-effective intervention in the primary care setting.

The lack of continuity of care and poor information sharing between primary and tertiary medical practitioners currently limits the effectiveness of ACP [40]. Technological innovations such as smart phone applications or digital wrist bracelets are potential vehicles to disseminate ACP information and documents during an emergency [38]. The recently-developed electronic health record in Australia also provides an ideal measure to carry ACP with the patients regardless of their location.

Despite using the best available evidence, a number of assumptions and simplification were necessary in our model. First, we estimated the age-dependent utility, mortality and end-of-life hospitalisation costs from the literature using multivariate regression analyses. Second, we assumed a constant rate of transition from mild to moderate to severe dementia, as well as stable preferences for end-of-life hospitalisation across disease state and age. We have performed extensive sensitivity analyses and highlighted influential factors (e.g. compliance rates and hospitalisation preferences). While these were strongly grounded on the existing literature, they should be revisited when new information becomes available. Lastly, we could only evaluate the ACP program using a health-system perspective, instead of a societal perspective. It is known that end-of-life care and medical decisions often impose a heavy burden, represented by potential productivity loss and a reduction in the mental and physical health of family and caregivers. While there are a large body of qualitative literature highlighting the burden, our literature search indicated that there were no reliable quantitative studies that could be used for this model. Additionally, if the (base case) ACP model was found to be cost-effective under the health-system perspective, which ignored the social costs *avoided* by family and caregivers if an individual has an ACP, a model with a social perspective would have a result that leads to the same cost-effective conclusion. The thresholds for ACP uptake and compliance rates and preferences for end-of-life hospitalisation, from a social perspective, would have been lower than those for the health system perspective.

## Conclusion

Our model indicates that a nationwide ACP intervention in the Australian primary care setting is a cost-effective option compared to usual care for the older population at risk of developing dementia. The result is largely driven by providing treatment and care consistent with patient ACP preferences, leading to fewer hospitalisations and less-intensive care at end-of-life. In order to meet the ACP uptake and compliance rates required in the model for ACP to be cost-effective, we recommend the Australian Government supports a national and consistent approach to ACP in the primary care setting.

Australia is ready to take the next step toward respecting end-of-life choices. Taking the initiative and time, and a positive attitude towards anticipating future scenarios seems universal across different parties and studies. All jurisdictions have developed regulatory and legislative frameworks to support ACP. There is also strong support for normalising ACP and promoting it earlier in life. Raising community awareness of ACP and adjusting attitudes towards ACP in primary care settings will enable the existing healthcare infrastructure to accommodate ACP, without a significant paradigm shift in service models. The ACP, which allows an individual to expressly state their wishes for end-of-life care, helps families, caregivers and health professionals to act, as far as possible, in accordance with the person’s own expressed and considered wishes and expectations.

## Additional file

Additional file 1: Data input and resources used in the economic model. (DOCX 70 kb)

## Abbreviations

ACD: Advance care document
ACP: Advance care planning
QALY: Quality adjusted life year
