# Supplementary material for: An economic model of advance care planning in Australia: a cost-effective way to respect patient choice
Source: BMC Health Serv Res. 2017 Dec 1;17:797. doi: 10.1186/s12913-017-2748-4 (PMC5709848; doi:10.1186/s12913-017-2748-4)
Supplement: Additional file 1: — Data input and resources used in the economic model. (DOCX 70 kb) [file 12913_2017_2748_MOESM1_ESM.docx]

**Supplementary File**

**Additional file 1: Data input and resources used in the economic model**

We conducted a thorough search in the Medline database and grey literature for relevant data to populate the model. In all instances, a basic search strategy was used with key words (and their combinations) such as advance care plan, advance directive, dementia, and Alzheimer’s disease. A manual search of the references of each identified article of interest was also completed for further information. Other sources of information included national epidemiological studies and costing reports. Summary of the main data inputs are presented in Tables A1 and A2.

**Transition probabilities**

The probability of developing dementia was estimated from the prevalence and incidence rate of dementia reported in the literature. Several parametric models (quadratic, exponential and power) were examined and the best fit (power regression) was used as the base case. Other estimates (quadratic and exponential) were used in the sensitivity analyses.

***Dementia progression***

There are numerous cost-effectiveness studies on treatments for dementia and Alzheimer’s disease [1]–[3]. The research on disease progression (from mild to moderate to severe) without a specific link to any treatment, on the other hand, is rare. The only relevant study identified was Neumann et al. (2001) that estimated transition probabilities of Alzheimer’s disease progression using the data from the Consortium to Establish a Registry for Alzheimer’s Disease (CERAD) [4]. CERAD is a longitudinal database of 1,145 patients with dementia at different severity stages who were examined annually by clinicians in 22 major medical centres in the United States between 1986 and 1995. This study accounted for age and gender differences, which were identified as important determinants of Alzheimer’s disease. Additionally, disease severity was defined more broadly, using the Clinical Dementia Rating instrument. Because of its large and diverse patient population and reliable annual assessment, CERAD was considered the most appropriate source for the estimation of transition probabilities. Since Alzheimer’s disease accounts for more than 80% of patients with dementia, these estimates were used to reflect the disease transition in the model.

***Coverage of advance care planning***

The probabilities of people aged over 65+ years having participated in advance care planning (ACP) were sourced from Detering et al. (2010), which is a randomised controlled trial studying the impact of ACP on end-of-life care [5]. In this study, 15% of the control group had an advance care plan. This figure is consistent with a study from South Australia that reported an ACP prevalence of 14% [6], and slightly higher than the estimate from a large survey (N=3,055) where the coverage of medical-related advance directives was below 13% [7].

The uptake rate if an ACP program is implemented is unknown. A meta-analysis [8] showed an increased likelihood of completing advance care plans in groups where ACP was introduced, compared to the control group (odd ratio: 3.26; 95% confidence interval 2.00 – 5.32). Some studies found that when offered, the uptake rate could be up to 80% [5] or as high as 99% [9]. However, the proportion of older adults who have a will, a relatively common legal document, was only 59% [6]. We conservatively used a 50% uptake rate as the base case. Various uptake rates of ACP were then tested to find the cost-effectiveness threshold.

The other transition probabilities were derived from the published literature and the age-dependent mortality risk was derived from the Australian life tables.

**Resources and costs**

Costs related to all healthcare resource items for the economic model were converted to annual values to accommodate the yearly cycle calculation. The currency is in Australian dollars, and converted to 2015 values using a currency conversion tool <http://eppi.ioe.ac.uk/costconversion/default.aspx>

***ACP program costs***

In accordance with best practice ACP, we propose a system-wide model with a flexible design that allows for the development and discussion of ACP in various settings, such as general practioners’ offices, nurse-led medical centres, residential care or homes. For instance, a GP can start the ACP discussion and distribute relevant material to their clients; the follow-up meetings can be conducted in the same GP office, led by an ACP-trained nurse, or at the client’s home with an ACP-trained social worker or Home Care Package case manager. It is estimated that 2-3 meetings, including the initial conversation, will be sufficient to discuss and finalise the ACP document. In the base case model, we conservatively allowed for one long meeting and three short meetings (a total of four meetings, compared to the likely three meetings suggested by clinicians and health professionals). The document can be revised in the future, and this is likely to happen around critical times such as when the individual is diagnosed with dementia, a terminal illness, or there are changes in life circumstances. We allowed for two revisions within the model (see Table A1 below). This means our proposed ACP package cost is between AU$670-AU$820 (for 4-6 meetings), from the initiation to the final revision. Sensitivity analyses were also conducted to allow for a smaller package (a public information session followed by two meetings, costing around AU$250) and a larger investment per individual (up to AUD$900).

It is estimated that the long discussion would contain information such as the patient’s history of experiences with death, understanding of illness, life goals, beliefs, communication patterns within the family and with their doctors, and financial and community resources. Each meeting can last between 60 and 90 minutes, which is equivalent to a comprehensive assessment and management plan for elderly patients (MBS item 141). Subsequent discussions, reviews and amendments are shorter appointments, which were assumed to be equivalent to a review of (treatment) management plans (MBS item 732).

***End-of-life cost***

During the end-of-life period, patients can be hospitalised or cared for in a hospice, residential care facility or at home. Hospital costs were sourced from Kardamanidis et al. (2007), which estimated hospital inpatient costs among older adults in New South Wales in the last year of life using a linked population database (sample of 70,384 people aged 65 years and over) [10]. The study noted that the hospital costs decreased with age, driven mostly by a relatively shorter length of stay by older people. We therefore incorporated the age-variantion in hospital costs in our model.

There is limited information on the end-of-life cost in other contexts, including home and community care, aged care facilities and hospices. While average daily cost per resident of residential aged care facility was available (approximately AU$250) [11], this cannot be used to calculate end-of-life care in this setting due to the lack of reliable information on end-of-life duration and additional palliative care costs. Our back-of-the-envelope calculation, assuming 20 days with a palliative care loading of 20% daily cost per resident, gave a cost of AU$6,000 per resident, which was approximately 40% of the average cost of end-of-life hospitalisation (approximately AU$14,000, average cost from Table A2).

Several studies have suggested that if the hospital was the default place for end-of-life care, the cost is generally higher than in other settings, including hospice and palliative facilities [12]–[14], while there is no significant improvement in mortality rates [12], [15], [16]. Additionally, evidence suggested that quality of life of hospitalised patients was not better than those who died at home, in an aged care facility or hospice [14], [17]. For the policy option, we examined various assumptions around the cost for patients who do not rely on hospitalisation for end-of-life care.

**Health state utility weights**

In our economic model, a quality-adjusted life year (QALY) was used to assess the extent of benefits gained from the interventions. A QALY is a metric that combines lifetime gained and the quality of the time gained. The theoretical underpinnings of QALYs relate to end-of-life decision making where people may prefer to give up some days or weeks at the end of their life in order to have a better quality of life in the months leading up to death. When QALYs are combined with the costs of providing the interventions, decision makers can understand their relative cost-effectiveness. In the case of scarce resources, interventions which provide the best incremental cost-effectiveness ratio should be reimbursed.

Quality of life was calculated using utility weights. For the normal health state (no dementia), we sourced the utility weights from Clemens et al. (2014), that estimated utility weights for Australians aged 18 and over [18]. We fitted various parametric functions to approximate the age-dependent weights. The quadratic function estimates proved to be the best fit and were used in the base case. Estimates by other functions were used in sensitivity analyses.

There is a large variation of weights applicable for end-of-life and dementia health states. For instance, patients with severe dementia were given a weight ranging from zero [19] to 0.427 [20]. For those with mild dementia, this range was from 0.37 [19] to 0.690 [21]. We combined these data in multivariate regressions to predict the marginal reductions in utility (or disutility) from the normal health state to mild, moderate or severe dementia and end of life. The age-dependent weights for each health state were then calculated by subtracting the respective disutility values from the utility weight of the normal health state. The calculation resulted in negative utility weights for very old people in the end-of-life health state. This is consistent with studies in the literature suggesting that there are health states worse than death (i.e. quality of life is less than zero) [22].

**Table S1: Key variables for the economic model**

| **Key variables** | **Values** | **Sources** |
| --- | --- | --- |
| **Probabilities** |  |  |
| Having advance care planning (ACP) |  |  |
| Current situation | 0.144 | White et al 2014 [23] |
| With intervention to increase ACP coverage | 0.500 | Assumption |
| Disease progression |  |  |
| Mild to mild | 0.614 | Neumann et al 2001 [4] |
| Mild to moderate | 0.364 | Neumann et al 2001 [4] |
| Moderate to moderate | 0.608 | Neumann et al 2001 [4] |
| Moderate to severe | 0.339 | Neumann et al 2001 [4] |
| Severe to severe | 0.847 | Neumann et al 2001 [4] |
| Die in hospital |  |  |
| Without ACP | 0.670 | Hunt et al 2013 [24] |
| With ACP | 0.150 | Hunt et al 2013 [24] |
| End-of-life preference and wish being respected |  |  |
| Without ACP | 0.296 | Detering et al 2010 [25] |
| With ACP | 0.862 | Detering et al 2010 [25] |
| Mortality risk | Age dependent | Australia life tables, see Table A2 below |
| Prevalence rate | Age dependent | Estimated from Clemen et al [18], Access Economics [26], see Table A2 below |
| **Costs** |  |  |
| Initial appointments for ACP (1 appointments) | $452.65 | Medical Benefit Scheme item 141 |
| Follow up appointment (3 appointments) and revision for ACP (2 revisions allowed) | $72.05 | Medical Benefit Scheme item 732 |
| End-of-life |  |  |
| In hospital | Age dependent | Estimated from Kardamanidis et al 2007 [10], see Table A2 below |
| Outside hospital, percentage of in hospital | 63%, age dependent | Assumption, based on Brumley et at 2007 [14], Shnoor et al 2007 [13], Caplan et al 2006 [12] |

**Table S2: Age-dependent transition utilities, probabilities and costs**

|  | **Utility weights** | | | | | **Probabilities** | | **Costs** |
| --- | --- | --- | --- | --- | --- | --- | --- | --- |
| **Age** | **Normal** | **Mild** | **Moderate** | **Severe** | **End-of-life** | **Dementia Incidence** | **Mortality** | **Hospital cost ($)** |
| 60 | 0.83240 | 0.58690 | 0.50950 | 0.28550 | 0.03090 | 0.00464 | 0.00542 | 23,760 |
| 61 | 0.83031 | 0.58481 | 0.50741 | 0.28341 | 0.02881 | 0.00542 | 0.00591 | 23,189 |
| 62 | 0.82824 | 0.58274 | 0.50534 | 0.28134 | 0.02674 | 0.00631 | 0.00644 | 22,627 |
| 63 | 0.82619 | 0.58069 | 0.50329 | 0.27929 | 0.02469 | 0.00733 | 0.00702 | 22,074 |
| 64 | 0.82416 | 0.57866 | 0.50126 | 0.27726 | 0.02266 | 0.00849 | 0.00767 | 21,529 |
| 65 | 0.82215 | 0.57665 | 0.49925 | 0.27525 | 0.02065 | 0.00982 | 0.00837 | 20,993 |
| 66 | 0.82016 | 0.57466 | 0.49726 | 0.27326 | 0.01866 | 0.01133 | 0.00916 | 20,465 |
| 67 | 0.81819 | 0.57269 | 0.49529 | 0.27129 | 0.01669 | 0.01305 | 0.01004 | 19,946 |
| 68 | 0.81624 | 0.57074 | 0.49334 | 0.26934 | 0.01474 | 0.01499 | 0.01103 | 19,433 |
| 69 | 0.81431 | 0.56881 | 0.49141 | 0.26741 | 0.01281 | 0.01719 | 0.01214 | 18,929 |
| 70 | 0.81240 | 0.56690 | 0.48950 | 0.26550 | 0.01090 | 0.01968 | 0.01341 | 18,431 |
| 71 | 0.81051 | 0.56501 | 0.48761 | 0.26361 | 0.00901 | 0.02247 | 0.01485 | 17,941 |
| 72 | 0.80864 | 0.56314 | 0.48574 | 0.26174 | 0.00714 | 0.02562 | 0.01651 | 17,457 |
| 73 | 0.80679 | 0.56129 | 0.48389 | 0.25989 | 0.00529 | 0.02916 | 0.01840 | 16,981 |
| 74 | 0.80496 | 0.55946 | 0.48206 | 0.25806 | 0.00346 | 0.03312 | 0.02053 | 16,510 |
| 75 | 0.80315 | 0.55765 | 0.48025 | 0.25625 | 0.00165 | 0.03756 | 0.02292 | 16,046 |
| 76 | 0.80136 | 0.55586 | 0.47846 | 0.25446 | -0.00014 | 0.04253 | 0.02563 | 15,588 |
| 77 | 0.79959 | 0.55409 | 0.47669 | 0.25269 | -0.00191 | 0.04807 | 0.02873 | 15,136 |
| 78 | 0.79784 | 0.55234 | 0.47494 | 0.25094 | -0.00366 | 0.05425 | 0.03229 | 14,690 |
| 79 | 0.79611 | 0.55061 | 0.47321 | 0.24921 | -0.00539 | 0.06113 | 0.03638 | 14,250 |
| 80 | 0.79440 | 0.54890 | 0.47150 | 0.24750 | -0.00710 | 0.06878 | 0.04106 | 13,815 |
| 81 | 0.79271 | 0.54721 | 0.46981 | 0.24581 | -0.00879 | 0.07728 | 0.04646 | 13,385 |
| 82 | 0.79104 | 0.54554 | 0.46814 | 0.24414 | -0.01046 | 0.08669 | 0.05266 | 12,961 |
| 83 | 0.78939 | 0.54389 | 0.46649 | 0.24249 | -0.01211 | 0.09712 | 0.05975 | 12,542 |
| 84 | 0.78776 | 0.54226 | 0.46486 | 0.24086 | -0.01374 | 0.10866 | 0.06783 | 12,128 |
| 85 | 0.78615 | 0.54065 | 0.46325 | 0.23925 | -0.01535 | 0.12141 | 0.07698 | 11,719 |
| 86 | 0.78456 | 0.53906 | 0.46166 | 0.23766 | -0.01694 | 0.13548 | 0.08726 | 11,315 |
| 87 | 0.78299 | 0.53749 | 0.46009 | 0.23609 | -0.01851 | 0.15098 | 0.09874 | 10,915 |
| 88 | 0.78144 | 0.53594 | 0.45854 | 0.23454 | -0.02006 | 0.16805 | 0.11148 | 10,520 |
| 89 | 0.77991 | 0.53441 | 0.45701 | 0.23301 | -0.02159 | 0.18683 | 0.12551 | 10,129 |
| 90 | 0.77840 | 0.53290 | 0.45550 | 0.23150 | -0.02310 | 0.20745 | 0.14048 | 9,743 |
| 91 | 0.77691 | 0.53141 | 0.45401 | 0.23001 | -0.02459 | 0.23009 | 0.15506 | 9,361 |
| 92 | 0.77544 | 0.52994 | 0.45254 | 0.22854 | -0.02606 | 0.25491 | 0.17050 | 8,983 |
| 93 | 0.77399 | 0.52849 | 0.45109 | 0.22709 | -0.02751 | 0.28209 | 0.19004 | 8,610 |
| 94 | 0.77256 | 0.52706 | 0.44966 | 0.22566 | -0.02894 | 0.31184 | 0.21229 | 8,240 |
| 95 | 0.77115 | 0.52565 | 0.44825 | 0.22425 | -0.03035 | 0.34435 | 0.23486 | 7,874 |
| 96 | 0.76976 | 0.52426 | 0.44686 | 0.22286 | -0.03174 | 0.37987 | 0.25570 | 7,512 |
| 97 | 0.76839 | 0.52289 | 0.44549 | 0.22149 | -0.03311 | 0.41861 | 0.27052 | 7,154 |
| 98 | 0.76704 | 0.52154 | 0.44414 | 0.22014 | -0.03446 | 0.46085 | 0.28541 | 6,799 |
| 99 | 0.76571 | 0.52021 | 0.44281 | 0.21881 | -0.03579 | 0.50686 | 0.30035 | 6,448 |
| 100+ | 0.76440 | 0.51890 | 0.44150 | 0.21750 | -0.03710 | 0.55693 | 0.31497 | 6,101 |

**References**

[1] C. Green, J. Picot, E. Loveman, A. Takeda, J. Kirby, and A. Clegg, ‘Modelling the cost effectiveness of cholinesterase inhibitors in the management of mild to moderately severe Alzheimer’s disease’, *Pharmacoeconomics*, vol. 23, no. 12, pp. 1271–1282, 2005.

[2] C. Green, J. Shearer, C. W. Ritchie, and J. P. Zajicek, ‘Model-Based Economic Evaluation in Alzheimer’s Disease: A Review of the Methods Available to Model Alzheimer’s Disease Progression’, *Value Health*, vol. 14, no. 5, pp. 621–630, Jul. 2011.

[3] A. Wimo *et al.*, ‘Health economic evaluation of treatments for Alzheimer′s disease: impact of new diagnostic criteria’, *J. Intern. Med.*, vol. 275, no. 3, pp. 304–316, Mar. 2014.

[4] P. J. Neumann *et al.*, ‘Measuring Alzheimer’s disease progression with transition probabilities Estimates from CERAD’, *Neurology*, vol. 57, no. 6, pp. 957–964, 2001.

[5] K. M. Detering, A. D. Hancock, M. C. Reade, and W. Silvester, ‘The impact of advance care planning on end of life care in elderly patients: randomised controlled trial.’, *BMJ*, vol. 340, p. c1345, 2010.

[6] B. White *et al.*, ‘Prevalence and predictors of advance directives in Australia’, *Intern. Med. J.*, vol. 44, no. 10, pp. 975–980, 2014.

[7] S. L. Bradley, R. J. Woodman, J. J. Tieman, and P. a Phillips, ‘Use of advance directives by South Australians: results from the Health Omnibus Survey Spring 2012’, *Med. J. Aust.*, vol. 201, no. 8, pp. 467–469, 2014.

[8] C. H. M. Houben, M. a. Spruit, M. T. J. Groenen, E. F. M. Wouters, and D. J. a Janssen, ‘Efficacy of advance care planning: A systematic review and meta-analysis’, *J. Am. Med. Dir. Assoc.*, vol. 15, no. 7, pp. 477–489, 2014.

[9] E. Ratner, L. Norlander, and K. McSteen, ‘Death at Home Following a Targeted Advance-Care Planning Process at Home: The Kitchen Table Discussion’, *J. Am. Geriatr. Soc.*, vol. 49, no. 6, pp. 778–781, 2001.

[10] K. Kardamanidis, K. Lim, C. Da Cunha, L. K. Taylor, and L. R. Jorm, ‘Hospital costs of older people in New South Wales in the last year of life’, *Med. J. Aust.*, vol. 187, no. 7, p. 383, 2007.

[11] Aged Care Financing Authority, ‘Fifth report on the Funding and Financing of the Aged Care Sector’, Australian Government, Canberra, Australia, Government Report, 2017.

[12] G. A. Caplan, A. Meller, B. Squires, S. Chan, and W. Willett, ‘Advance care planning and hospital in the nursing home’, *Age Ageing*, vol. 35, pp. 581–585, 2006.

[13] Y. Shnoor, M. Szlaifer, A. S. Aoberman, and N. Bentur, ‘The Cost of Home Hospice Care for Terminal Patients in Israel’, *Am. J. Hosp. Palliat. Med.*, vol. 24, no. 4, pp. 284–290, Aug. 2007.

[14] R. Brumley *et al.*, ‘Increased Satisfaction with Care and Lower Costs: Results of a Randomized Trial of In-Home Palliative Care’, *J. Am. Geriatr. Soc.*, vol. 55, no. 7, pp. 993–1000, Jul. 2007.

[15] K. S. Boockvar, A. L. Gruber-Baldini, L. Burton, S. Zimmerman, C. May, and J. Magaziner, ‘Outcomes of Infection in Nursing Home Residents with and without Early Hospital Transfer’, *J. Am. Geriatr. Soc.*, vol. 53, no. 4, pp. 590–596, Apr. 2005.

[16] T. R. Fried, M. R. Gillick, and L. A. Lipsitz, ‘Short-Term Functional Outcomes of Long-Term Care Residents with Pneumonia Treated with and without Hospital Transfer’, *J. Am. Geriatr. Soc.*, vol. 45, no. 3, pp. 302–306, Mar. 1997.

[17] M. M. Garrido, T. A. Balboni, P. K. Maciejewski, Y. Bao, and H. G. Prigerson, ‘Quality of Life and Cost of Care at the End of Life: The Role of Advance Directives’, *J. Pain Symptom Manage.*, vol. 49, no. 5, pp. 828–835, May 2015.

[18] S. Clemens, N. Begum, C. Harper, J. A. Whitty, and P. A. Scuffham, ‘A comparison of EQ-5D-3L population norms in Queensland, Australia, estimated using utility value sets from Australia, the UK and USA’, *Qual. Life Res.*, vol. 23, no. 8, pp. 2375–2381, Oct. 2014.

[19] P. M. McMahon, S. S. Araki, E. A. Sandberg, P. J. Neumann, and G. S. Gazelle, ‘Cost-Effectiveness of PET in the Diagnosis of Alzheimer Disease’, *Radiology*, vol. 228, no. 2, pp. 515–522, Aug. 2003.

[20] A. Wimo, B. Mattson, I. Krakau, T. Eriksson, A. Nelvig, and G. Karlsson, ‘Cost-utility analysis of group living in dementia care’, *Int. J. Technol. Assess. Health Care*, vol. 11, no. 1, pp. 49–65, 1995.

[21] A. R. Willan, R. Goeree, E. M. Pullenayegum, C. McBurney, and G. Blackhouse, ‘Economic Evaluation of Rivastigmine in Patients with Parkinson’s Disease Dementia’, *Pharmacoeconomics*, vol. 24, no. 1, pp. 93–106, 2006.

[22] P. A. Scuffham and M. J. Taylor, ‘Economics and decisions to end life: van Acht and Stooker revisited’, *Appl. Health Econ. Health Policy.*, vol. 1, no. 3, pp. 1–6, 2002.

[23] B. White *et al.*, ‘Prevalence and predictors of advance directives in Australia: Advance directives in Australia’, *Intern. Med. J.*, vol. 44, no. 10, pp. 975–980, Oct. 2014.

[24] R. Hunt, M. Seal, and L. Owen, ‘Estimating impact of ACP activity on hospital admission, OBD and acute care savings’, presented at the ACPEL Conference, Melbourne, 2013.

[25] K. M. Detering, A. D. Hancock, M. C. Reade, and W. Silvester, ‘The impact of advance care planning on end of life care in elderly patients: randomised controlled trial’, *BMJ*, vol. 340, no. mar23 1, pp. c1345–c1345, Mar. 2010.

[26] Access Economics, ‘Dementia Across Australia 2011-2050.pdf’, Deloitte Access Economics Pty Ltd, 2011.
